# Defining the function of SUMO system in pod development and abiotic stresses in Peanut

**DOI:** 10.1186/s12870-019-2136-9

**Published:** 2019-12-29

**Authors:** Yiyang Liu, Jiao Zhu, Sheng Sun, Feng Cui, Yan Han, Zhenying Peng, Xuejie Zhang, Shubo Wan, Guowei Li

**Affiliations:** 1 Biotechnology Research Center, Shandong Academy of Agricultural Sciences; Shandong Provincial Key Laboratory of Crop Genetic Improvement, Ecology and Physiology, Jinan, China; 2 College of Life Science, Shandong Normal University, Jinan, China; 3 Shandong Key Laboratory of Greenhouse Vegetable Biology, Institute of Vegetables and Flowers, Shandong Academy of Agricultural Sciences, Jinan, China; 4 College of Teacher Education, Heze University, Heze, China

**Keywords:** Peanut, SUMO pathway, SUMOylation, Pod development, Abiotic stresses

## Abstract

**Background:**

Posttranslational modification of proteins by small ubiquitin like modifier (SUMO) proteins play an important role during the developmental process and in response to abiotic stresses in plants. However, little is known about SUMOylation in peanut (*Arachis hypogaea* L.), one of the world’s major food legume crops. In this study, we characterized the SUMOylation system from the diploid progenitor genomes of peanut, *Arachis duranensis* (AA) and *Arachis ipaensis* (BB).

**Results:**

Genome-wide analysis revealed the presence of 40 SUMO system genes in *A. duranensis* and *A. ipaensis*. Our results showed that peanut also encodes a novel class II isotype of the SCE1, which was previously reported to be uniquely present in cereals. RNA-seq data showed that the core components of the SUMOylation cascade *SUMO1/2* and *SCE1* genes exhibited pod-specific expression patterns, implying coordinated regulation during pod development. Furthermore, both transcripts and conjugate profiles revealed that SUMOylation has significant roles during the pod development. Moreover, dynamic changes in the SUMO conjugates were observed in response to abiotic stresses.

**Conclusions:**

The identification and organization of peanut SUMO system revealed SUMOylation has important roles during stress defense and pod development. The present study will serve as a resource for providing new strategies to enhance agronomic yield and reveal the mechanism of peanut pod development.

## Background

Posttranslational modifications (PTMs) set a reversible mark on specific amino acids, allowing dynamic and reversible changes to protein function [1, 2]. Among these, the small ubiquitin-like modifier (SUMO) peptide to protein substrates (SUMOylation) is a major posttranslational regulatory process in eukaryotes [3]. The process of SUMOylation is illustrated in four steps analogous to the E1-E3 stages of ubiquitinoylation. First, SUMO precursors are processed by SUMO-specific cysteine (Cys) proteases (ULP, ubiquitin-like protein protease) with SUMO peptidase activity that carboxyl terminally truncates the preprotein to the diglycine (GG) motif. The SUMO carboxyl-terminal G is linked to AMP (SUMO-AMP) that is catalyzed by the heterodimeric E1 SUMO activating enzymes 1 and 2 (SAE1 and SAE2) in an ATP-dependent reaction. Subsequently, activated SUMO is transferred to the active site Cys of the SUMO conjugating enzyme (SCE). SCE can conjugate SUMO to the substrate proteins, resulting in the formation of an isopeptide linkage between the carboxyl-terminal Gly of SUMO and the ε-amino group of a lysine (Lys) residue within the substrate [1]. The SUMO ligases play important roles in vivo to determine substrate specificity and the extent of SUMOylation [4]. The reversibility of SUMO conjugation results from the hydrolysis of the isopeptide bond by SUMO-specific proteases to release SUMO for further conjugation cycles [5].

SUMOylation plays an essential role in plant development and stresses [6]. Within the SUMO system, *sae1*, *sae2*, and *sce1* single mutants, and *sumo1 sumo2* and *siz1 mms21* double mutants are embryo lethal [7, 8], which confirms that the core conjugation cascade is essential in Arabidopsis, as it is in animals [9]. The *spf1 spf2* ULP double mutants exhibit reduced seed set and self-fertilization [10, 11]. A similar phenomenon is observed in *ots1 ots2* ULP mutants [12, 13]. Studies using loss-of-function mutants of E3 ligase have identified many SUMO substrates involved in nutrient homeostasis, signaling by hormones (gibberellins (GA), SA, abscisic acid (ABA)), light-sensing, stem cell maintenance, and the photoperiodic control of flowering [14–19]. On the other hand, SUMO has long been established as a strong player in the plant stress system [20–22]. In Arabidopsis, it is well established that the abundance of SUMO conjugates increases in response to different abiotic stresses such as high salinity, high temperature, freezing, drought, and oxidative stress [13, 23–28]. Recently, numerous potential SUMOylation targets involved in abiotic stresses were identified using proteomics [23, 29, 30].

Cultivated peanut (*Arachis hypogaea* L.) is an important oil crop for human nutrition and grown world widely [31]. Peanut is allotetraploid (AABB, 2n = 4*x* = 40) originated from a single hybridization event between *Arachis. duranensis* (AA genome) and *Arachis. ipaensis* (BB genome), and subsequently underwent spontaneous genome duplication [32, 33]. The unique feature of peanut fruit development is that the fertilization occurs in the flowers above the ground but the fruits develop below the ground. Following fertilization, an intercalary meristem at the base of the ovary undergoes active division leading to a pointed stalk-like structure called the “peg” [34]. After the peg penetrates into the soil, the end of the peg expands to form the “pod”. Then, the pod develops and a mature peanut pod is produced [35, 36]. The subterranean fructification is the most prominent characteristic of seed production in peanut and thus has the biologically important for studying organogenesis and evolution [37]. Additionally, peanut often cultivated in the semiarid tropical regions, are often exposed to water stress (mid-season and end-season) and high temperature (> 34 °C) during the critical stages of flowering and pod development [38]. And, low-salinity fields were attempted to apply for peanut cultivation to gain more crop production in China in recent years [39, 40], which made peanut have to suffer to salt stress.

Previous studies in Arabidopsis and the crop species indicated that protein SUMOylation plays a key role during plant developmental process or in response to various abiotic stresses [1, 9, 41–44]. However, no available information of SUMOylation in peanut has been reported, especially, SUMOylation variation with the pod development. So, it is considerably interesting to comprehensively characterize SUMO system in peanut. In the present study, the core components of SUMO system were identified and the phylogenetic analysis were presented in peanut. The tissue-specific expression pattern of SUMO system genes and SUMOylation variation were correlative analyzed during pod development. Moreover, the SUMOylation profiles in response to heat, drought, salt and H_2_O_2_ stresses were further investigated. Our results indicated that SUMOylation played a potential role in pod development and abiotic stress response and would favor further studies on SUMOylation substrates identification in peanut.

## Results

### Identification and characterization of Peanut SUMO pathway genes

**Fig. 1:**
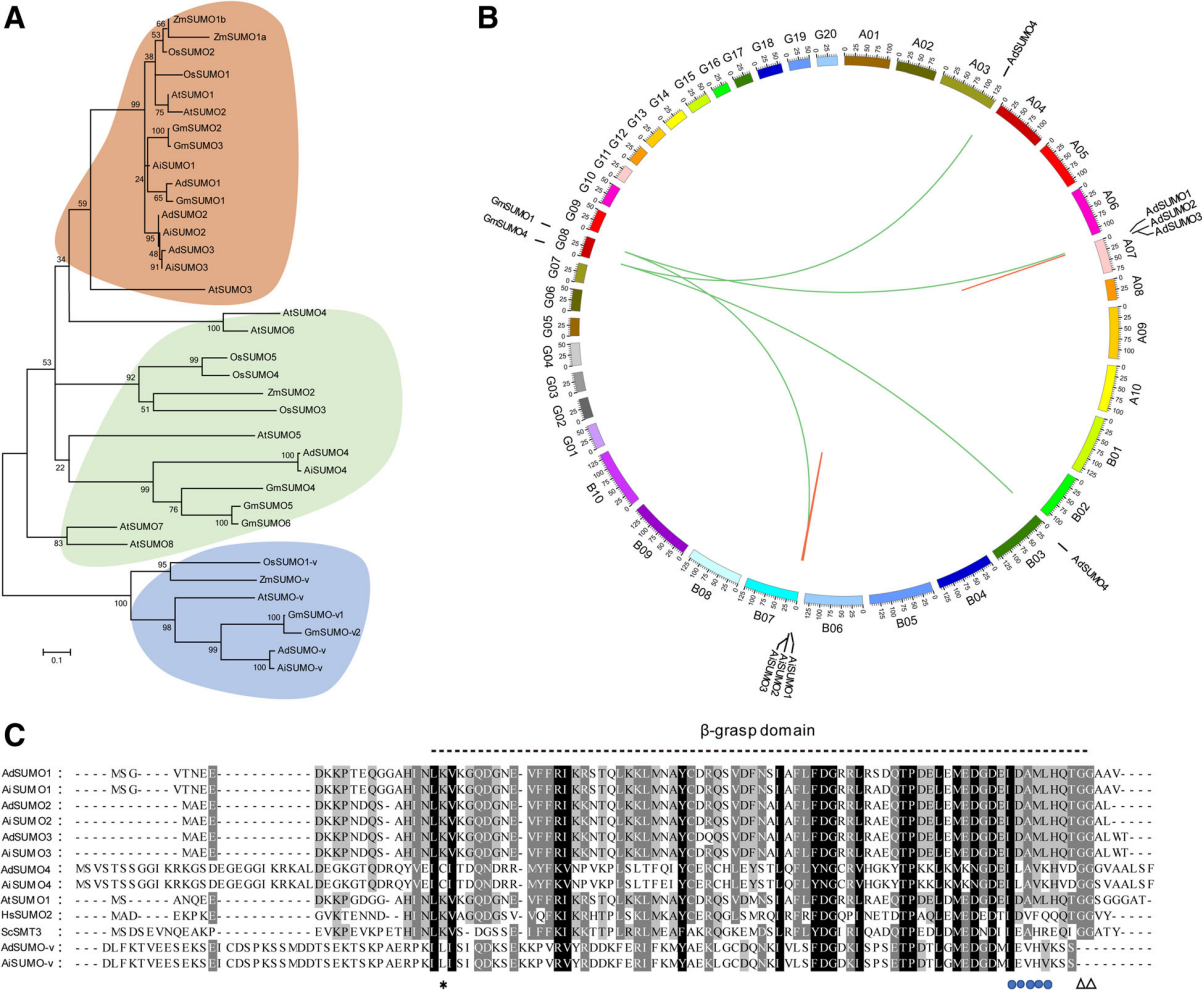
SUMO-related proteins in peanut and other plant species. **a** Protein sequences from *Arabidopsis thaliana*, *Glycine max*, *Oryza sativa*, Peanut and *Zea mays* were used to construct the phylogenetic tree by the neighbor-joining method in MEGA 5. They were classified into three groups: canonical SUMO, non-canonical SUMO, and SUMO-variant. **b** Synteny analyses of SUMOs in peanut and *Glycine max*. The short light black lines on the circle indicate the approximate chromosome location of peanut or *Glycine max* SUMOs. Syntenic regions between peanut and *Glycine max* SUMOs are represented by colored lines. **c** Alignment of SUMO sequence reveals conserved and divergent residues from peanut, Arabidopsis (AtSUMO1), human (HsSUMO2, NP_008868.3), and yeast (ScSmt3, KZV12750.1). Only conserved region is shown. The black dotted line locates the β-grasp-fold. The black triangle locates the processing site by ULP that exposes the diGly motif essential for conjugation in canonical SUMOs. The asterisk denotes the conserved Lys required for forming SUMO-chains. The blue circle dots denote SUMO interacting motif (SIMs). Gray and black boxes identify similar and conserved amino acids, respectively. Dashes denote gaps. At, *Arabidopsis thaliana*; Zm, *Zea mays*; Os, *Oryza sativa*; Gm, *Glycine max*; Hs, *Homo sapiens*; Sc, *Saccharomyces cerevisiae*

Whole genome sequencing of the two ancestral species (*Arachis. duranensis* and *Arachis. ipaensis*) has been completed [32]. First, we scanned the genome of the diploid ancestors: *Arachis. duranensis* (AA genome) and *Arachis. ipaensis* (BB genome) available in PeanutBase (http://peanutbase.org/) using BLASTP and TBLASTN. The amino acid sequences of the known Arabidopsis SUMO, E1, E2, E3, and SUMO protease protein were used as queries. Due to the still incomplete assembly of the genome, a number of initially identified loci had incorrectly assigned junctions. These assemblies were then corrected by aligning the genomic sequences to the corresponding transcripts composed by focused reverse transcription (RT)-PCR analyses with peanut total RNA. A total of 40 genes encoding the core components of the SUMO pathway were identified from two wild peanut species (Table 1), and the gene structure was analyzed (Additional file 1: Figure S1). Our list showed that each of the AA and BB genomes contain four SUMO genes, designated as *AdSUMO1* to *AdSUMO4* and *AiSUMO1* to *AiSUMO4*, respectively. The SUMO genes which have the homologous loci between AA and BB genomes had consistent amino acid sequences (Additional file 2: Figure S2). To further understand the evolutionary relationship between these SUMO isoforms, a phylogenetic tree was constructed together with homologues from other plant genomes (Fig. 1a). The phylogenetic analysis revealed a highly conserved SUMO group called as canonical group including AdSUMO1/2/3, AiSUMO1/2/3, GmSUMO1/2/3, AtSUMO1/2, ZmSUMO1a/b and OsSUMO1/2. In contrast, the “noncanonical” group included AdSUMO4, AiSUMO4, GmSUMO4/5/6, AtSUMO3/5 shared lower amino acid identity to the canonical group. Amino acid sequence alignment found that non-canonical members also had the C-terminal di-Gly motif necessary for conjugation (Fig. 1b), which indicated their potential ability of covalently attach to the target proteins. To support this finding, we identified some residues that are important for the non-covalent binding of SUMOs to E1, E2, and SUMO-interacting motifs (SIMs, ψψXψD/S/E, D/S/EψXψψ or ψψDLT, where ψ stands for the hydrophobic amino acids and X represents any residue) that are conserved across the biological kingdoms [45] (Fig. 1b).

**Table 1:** Identification of peanut SUMO pathway genes

Group	Gene name	Gene ID	Chromosome location	Gene length (bp)	Number of Exon	Protein length (aa)	MW (kDa)
SUMO	*AdSUMO1*	*Aradu.1V55H*	Aradu.A07:2519393..2521789	297	3	98	10.99
	*AdSUMO2*	Aradu.TI81R.1*	Aradu.A07:7003804..7006237	279	3	92	10.50
	*AdSUMO3*	Aradu.TI81R.2*	Aradu.A07:7006238..7008635	285	3	94	10.76
	*AdSUMO4*	*Aradu.453WH*	Aradu.A03:133838894..133842403	366	3	121	13.54
	*AiSUMO1*	*Araip.7CF1P*	Araip.B07:2253259..2255182	297	3	98	10.98
	*AiSUMO2*	Araip.K6C0S.1*	Araip.B07:6624978..668690	291	3	96	10.50
	*AiSUMO3*	Araip.K6C0S.2*	Araip.B07:6628691..6631840	285	3	94	10.79
	*AiSUMO4*	*Araip.I6MT4*	Araip.B03:651269..654312	366	3	121	13.54
	*AdSUMO-v*	*Aradu.PIA7H*	Aradu.A09:113452632..113454825	648	6	215	24.40
	*AiSUMO-v*	*Araip.FUS30*	Araip.B09:143973304..143975754	648	6	215	24.40
E1	*AdSAE1a*	*Aradu.1407H*	Aradu.A06:4758850..4762956	972	10	323	35.88
	*AdSAE1b*	*Aradu.RI74K*	Aradu.A03:104823784..104827358	684	7	227	25.33
	*AiSAE1*	*Araip.451UD*	Araip.B06:13340380..13344180	900	10	299	33.25
	*AdSAE2*	*Aradu.BX3KE*	Aradu.A08:42379801..42386216	1539	13	512	56.75
	*AiSAE2*	*Araip.M37G9*	Araip.B08:119362966..119369313	1860	11	626	68.87
E2	*AdSCE1a*	*Aradu.RBV6Z*	Aradu.A01:14503933..14509065	480	5	159	18.00
	*AdSCE1b*	*Aradu.2B732*	Aradu.A06:110697865..110698972	321	3	106	12.08
	*AiSCE1a*	*Araip.66GXB*	Araip.B01:19606726..19610692	480	5	159	18.00
	*AiSCE1b*	*Araip.WG1GW*	Araip.B06:135477520..135478857	366	3	121	13.58
	*AiSCE1c*	*Araip.I7QC8*	Araip.B04:76532164..76536153	414	5	137	15.40
E3	*AdSIZ1a*	*Aradu.RH9NQ*	Aradu.A03:1661304..1669701	2724	17	907	98.73
	*AdSIZ1b*	*Aradu.2M17J*	Aradu.A08:26107744..26116974	3132	19	1043	114.21
	*AiSIZ1a*	*Araip.K7FTA*	Araip.B03:3622548..3632024	2901	20	966	105.17
	*AiSIZ1b*	*Araip.PE4S7*	Araip.B08:3460391..3468068	2631	17	876	95.26
	*AdMMS21*	*Aradu.3IM1W*	Aradu.A08: 38168068..38171890	624	6	207	23.68
	*AiMMS21a*	*Araip.A34IS*	Araip.B08:25183279..25186758	540	5	179	20.43
	*AiMMS21b*	*Araip.C4BMV*	Araip.B07:113470811..113474288	636	6	211	23.68
	*AdPIAL1*	*Aradu.JZV9T*	Aradu.A05:14967345..14976725	2934	18	977	107.72
	*AiPIAL1*	*Araip.QU734*	Araip.B05:15738434..15746964	2793	16	930	102.43
SUMO protease	*AdELS1*	*Aradu.JT96L*	Aradu.A04:17811171..17816532	1620	9	539	61.58
	*AdELS2*	*Aradu.GFZ76*	Aradu.A01:96559057..96563010	1323	9	440	50.47
	*AiELS1*	*Araip.67K5R*	Araip.B04:17589501..17594881	1620	9	539	61.75
	*AiELS2*	*Araip.D5WZP*	Araip.B01:130135441..130140134	1395	9	464	53.26
	*AdOTS1*	*Aradu.82WQQ*	Aradu.A03:124642019..124648824	1734	14	577	65.63
	*AiOTS1*	*Araip.DF1J8*	Araip.B03:125248584..125255659	1914	15	637	71.80
	*AdFUG1*	*Aradu.FF6JW*	Aradu.A03:39617743..39621085	864	7	287	33.79
	*AiFUG1*	*Araip.JJ6Y2*	Araip.B10:1739374..1742213	726	2	241	28.46
	*AiFUG2*	*Araip.70UIF*	Araip.B08:129570462..129573726	867	7	288	33.61
	*AdSPF1*	*Aradu.BN4MX*	Aradu.A08:14475447..14483401	2952	16	983	113.07
	*AiSPF1*	*Araip.9HI6M*	Araip.B07:122679114..122687193	2871	16	956	107.18

*bp* base pair, *aa* amino acids, *MW* molecular weight, *kDa* kilo dalton, The asterisk denotes the genes which predicted one gene by genome annotation, are actually two tandem repeat genes identified by DNA sequence

Synteny analysis was preformed between the peanut and soybean sequences to gain further understanding of the origin and evolutionary relationship of the SUMOs (Fig. 1b). Four orthologous gene pairs identified are as follows: *AdSUMO1*-*GmSUMO1*, *AiSUMO1*-*GmSUMO1*, *AdSUMO4*-*GmSUMO4*, and *AiSUMO4*-*GmSUMO4*. Only two paralogous pairs of *SUMO* gene were found in peanut: *AdSUMO2*-*AdSUMO3* and *AiSUMO2*-*AiSUMO3*. The ortholog suggests that *SUMO1* and *SUMO4* genes in peanut and soybean may descended from a common evolutionary ancestor. The genes *SUMO2* and *SUMO3* in peanut plant are likely to have risen from a recent tandem duplication event, given that their loci onto peanut chromosomes are next each other.

Besides the canonical and the noncanonical group, a *SUMO-v* family was widely present in the plant genome that was conserved within the predicted β-grasp fold, and lacked an obvious C-terminal diGly motif [43]. Sequence alignment identified two longer SUMO related proteins in the peanut genome belonging to the *SUMO-v* family, and were designated as *AdSUMO-v* and *AiSUMO-v*. Phylogenetic analysis also divided *AdSUMO-v* and *AiSUMO-v* into SUMO-*v* groups with paralogs from other species. *Ad/AiSUMO*-*v* had the N-terminal extension of more than 100 residues upstream of the SUMO β-grasp domain without an exposed diGly motif (Additional file 2: Figure S2). Thus, *SUMO-v* might not involve in the well-studied E1/E2/E3 SUMOylation pathway, but probably had other functions to be explored in plants.

In peanut, there are three subunit SAE1 isoforms (AdSAE1a/b and AiSAE1) and two subunit SAE2 isoforms (Ad/AiSAE2) (Table 1). The phylogenetic analysis of SAE from various plant species showed that AdSAE1a/b and AiSAE1 were evolutionarily most closely to GmSAE1a/b, and SAE2 had high similarities with GmSAE2 (Additional file 3: Figure S3).

**Fig. 2:**
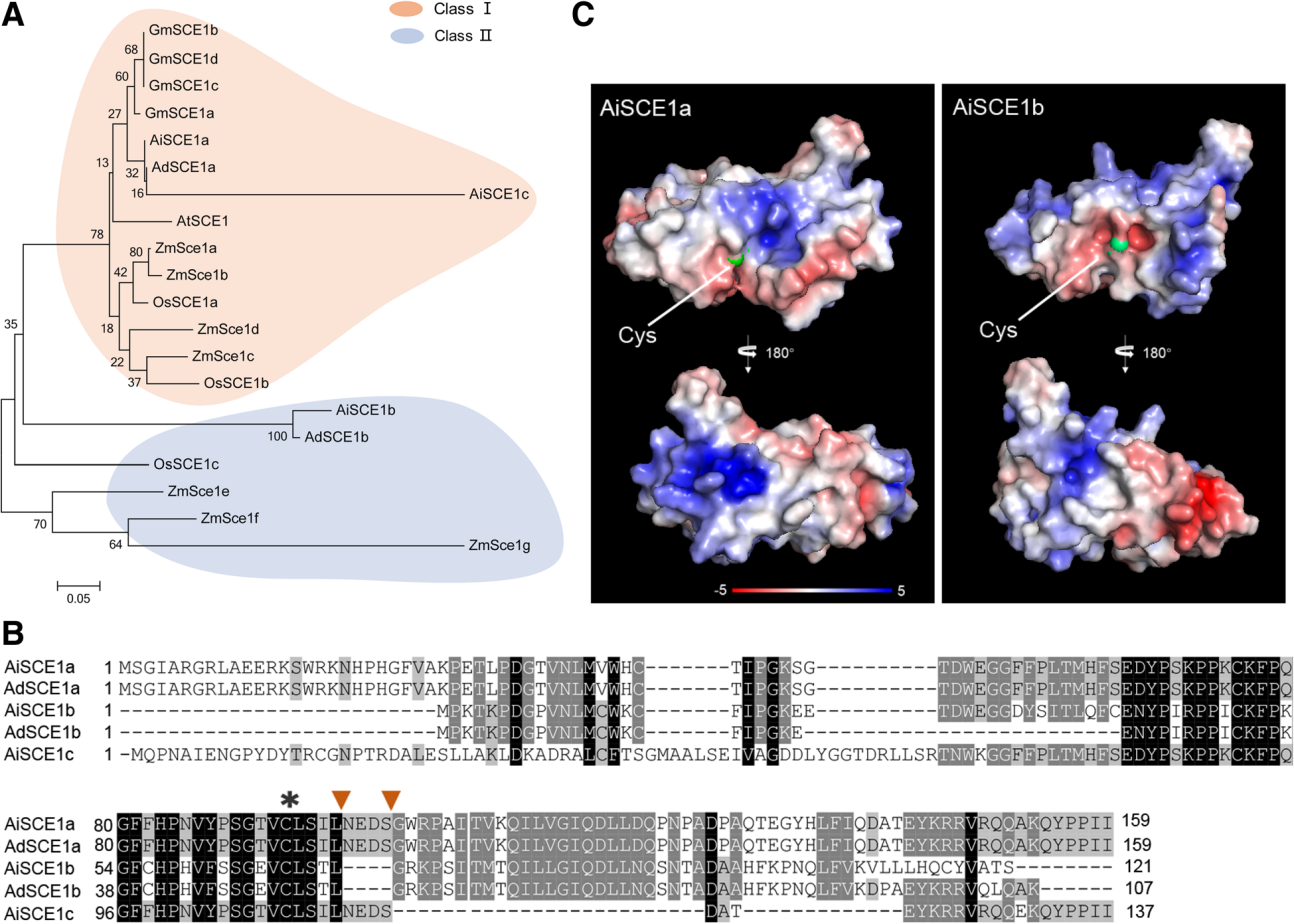
Peanut encode a novel SCE1 subfamily. **a** Phylogenetic analysis of SCE1 sequences. The phylogenetic relationship of SCE1 protein sequences from *Arabidopsis thaliana* (At), *Zea mays* (Zm), *Oryza sativa* (Os), *Glycine max* (Gm) and Peanut was assessed by the neighbor-joining method using MEGA 5. The scale bar indicates the P distance. **b** Alignment sequences of Ad/AiSCE1a, Ad/AiSCE1b, AiSCE1c and Arabidopsis AtSCE1. The asterisk highlights the active-site Cys. Brown arrowheads located residues indicates an amino acid deletion in AiSCE1b and AdSCE1b. Gray and black boxes indicate similar and conserved amino acids, respectively. Dashes denote gaps. **c** Electrostatic surface charges surrounding the active-site Cys (green) are distinct between AiSCE1a and AiSCE1b. Surface electrostatics shown in two orientations were calculated using the Adaptive Poisson-Bolzmann Solver plugin in PyMOL. Blue indicates positive charges and red indicates negative charges

In the second step of the pathway, SUMO is transferred from the E1 to the active site Cys of the E2 (SCE), forming a SUMO-E2 thioester intermediate [20]. The peanut genome contains five SCE genes, including *AdSCE1a/b* in the AA genome and *AiSCE1a/b/c* in the BB genome. To further understand the origins of these E2 genes, we collected several SCE1 proteins that were previously reported from other plant genomes and constructed a phylogenetic tree (Fig. 2a). The phylogenetic analysis revealed the presence of two potential subfamilies: one including *AdSCE1a*, *AiSCE1a*, and *AiSCE1c* genes that closely related to AtSCE1, and the other subfamily included *AdSCE1b* and *AiSCE1b* that closely related to the maize *SCE1e* to *SCE1g* genes. Interestingly, the results were consistent with previous classification in the cereal branch of monocots which divided E2s into two distinct subfamilies that were designated as class I and class II. Class I representatives were found in all eukaryotes and essential members such as Arabidopsis SCE1 and yeast Ubc9. In contrast, the class II clade was found only in the cereal branch of monocots such as maize SCE1e to SCE1g [43]. The class II clade had a more negative electrostatic surface around the active-site Cys in addition to an invariant deletion of an amino acid near the active-site pocket [43]. Given that SCE1b of dicotyledonous peanut belongs to the class II clade by the phylogenetic analysis, we further aligned the amino acid sequences of SCE1b with SCE1a and SCE1c. The results showed that, similar to the members of class II group in maize, Ad/AiSCE1b also had numerous nonconservative substitutions, along with an invariant deletion of an amino acid near the active-site Cys (Fig. 2b). To further confirm whether peanut SCE1b belonged to the class II group or not, we predicted the three-dimensional structures of AiSCE1a (class I) and AiSCE1b (class II) by SWISS-MODEL using the available human UBC9 model as a template, and analyzed electrostatic surface charges. The affected surface in AiSCE1a, similar to AtSCE1, was enriched in positively charged residues surrounding the active-site Cys, whereas AiSCE1b was enriched with negatively charged residues around the active-site Cys (Fig. 2c). These results suggested that apart from the cereal branch of monocots, the class II group of E2 proteins were also present in the dicotyledonous peanut. Additionally, the amino acid sequence alignment revealed that the sequences of AdSCE1a and AiSCE1a were completely identical, indicating that SCE1a is highly conserved in peanut evolution and is essential for peanut development (Additional file 4: Figure S4).

**Fig. 3:**
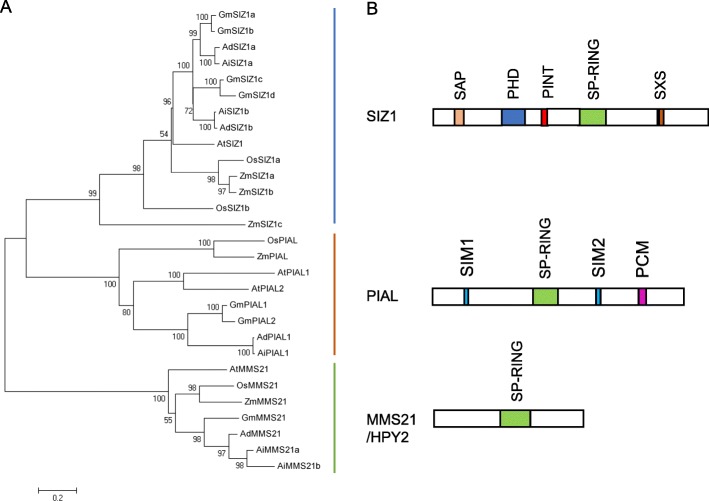
The phylogenetic tree and schematic representation of three types of E3 ligase in peanut. **a** E3 protein sequences from *Arabidopsis thaliana* (At), *Zea mays* (Zm), *Oryza sativa* (Os), *Glycine max* (Gm), and peanut were used to construct the phylogenetic tree. These E3 ligases are classified into SIZ1, PIAS and HPY2/MMS21 groups. **b** Schematic representation of functional domains of SIZ1, PIAS and HPY2/MMS21type SUMO ligase

SUMO E3 ligases are E2-interacting proteins that facilitate SUMO from SCE1 transfer to substrates in vivo, although some substrates do not require the presence of E3 ligases to be modified in vitro [9]. The SUMO ligases identified in Arabidopsis, including SIZ1, MMS21 and PIAL1/2, belong to the SP-RING family [9]. SUMO E3 ligases (E3) in peanut also can be divided into three subfamilies: SIZ1, PIAL, and MMS21 (Fig. 3). The peanut SIZ1 family includes four proteins (AdSIZ1a/b and AiSIZ1a/b), which contain the conserved SAP, PHD, MIZ/SP-RING domain, PINIT and SXS motifs (Additional file 5: Figure S5). Phylogenetic analysis showed that the Ad/AiSIZ1a were most evolutionarily related to GmSIZ1a/b, and Ad/AiSIZ1b were most close to GmSIZ1c/d. There were only two PIAL homologues (Ad/AiPIAL1) identified in the AA and BB genomes. Sequence alignment showed the peanut PIALs have the MIZ/SP-RING domain as well as PCM and SIM motifs (Additional file 6: Figure S6). Phylogenetic analysis showed that Ad/AiPIAL1 were evolutionarily most closely to GmPIAL1 (Fig. 3). Three homologues of AtMMS21 including AdMMS21a and AiMMS21a/b were identified which contain the conserved SP-RING domain (Additional file 7: Figure S7). There are two *MMS21* copies in AA genome and one *MMS21* copy in BB genome. Amino acid alignments revealed that AiMMS21a/b showed more divergence with AdMMS21a at the N-terminus, implying that MMS21 protein in the two sub-genomes of peanut might have taken different evolutionary routes (Fig. 3).

**Fig. 4:**
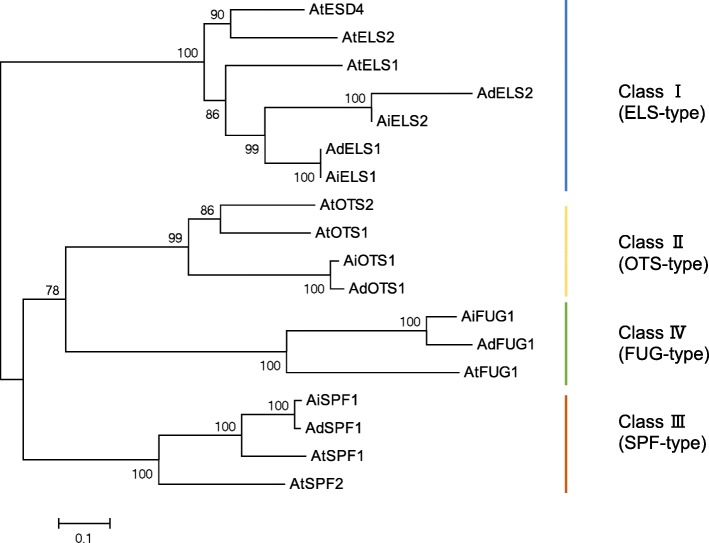
Phylogenetic relationships and subgroup designations of SUMO protease in peanut genome. SUMO protease sequences from *Arabidopsis thaliana* (At) and peanut were used to construct the phylogenetic tree. These SUMO proteases are classified into four type groups (ELS, OTS, FUG, and SPF). The phylogenetic analysis was carried out using MEGA 5

Plant deSUMOylating proteases belonging to the Ub-Like Protease (ULP) C48 gene family control deconjugation and SUMO maturation [5]. Using SUMO proteases in Arabidopsis as queries, 11 SUMO proteases were identified in peanut genome. The SUMO protease genes in peanut could be classified into four groups: Class I ELS-type, Class II OTS-type, Class III FUG-type, and Class IV SPF-type (Fig. 4), which were consistent with the classification results in Arabidopsis [46, 47]. Furthermore, all of them were found to have a peptidase_C48 domain (Additional file 8: Figure S8), inferring similar SUMO protease activity.

### Transcriptional analysis of the SUMO system

To further understand the spatiotemporal expression of SUMO system in peanut, the expression pattern of the associated genes was analyzed from the RNA-seq data sets from 22 tissue samples that previously investigated [48].

**Fig. 5:**
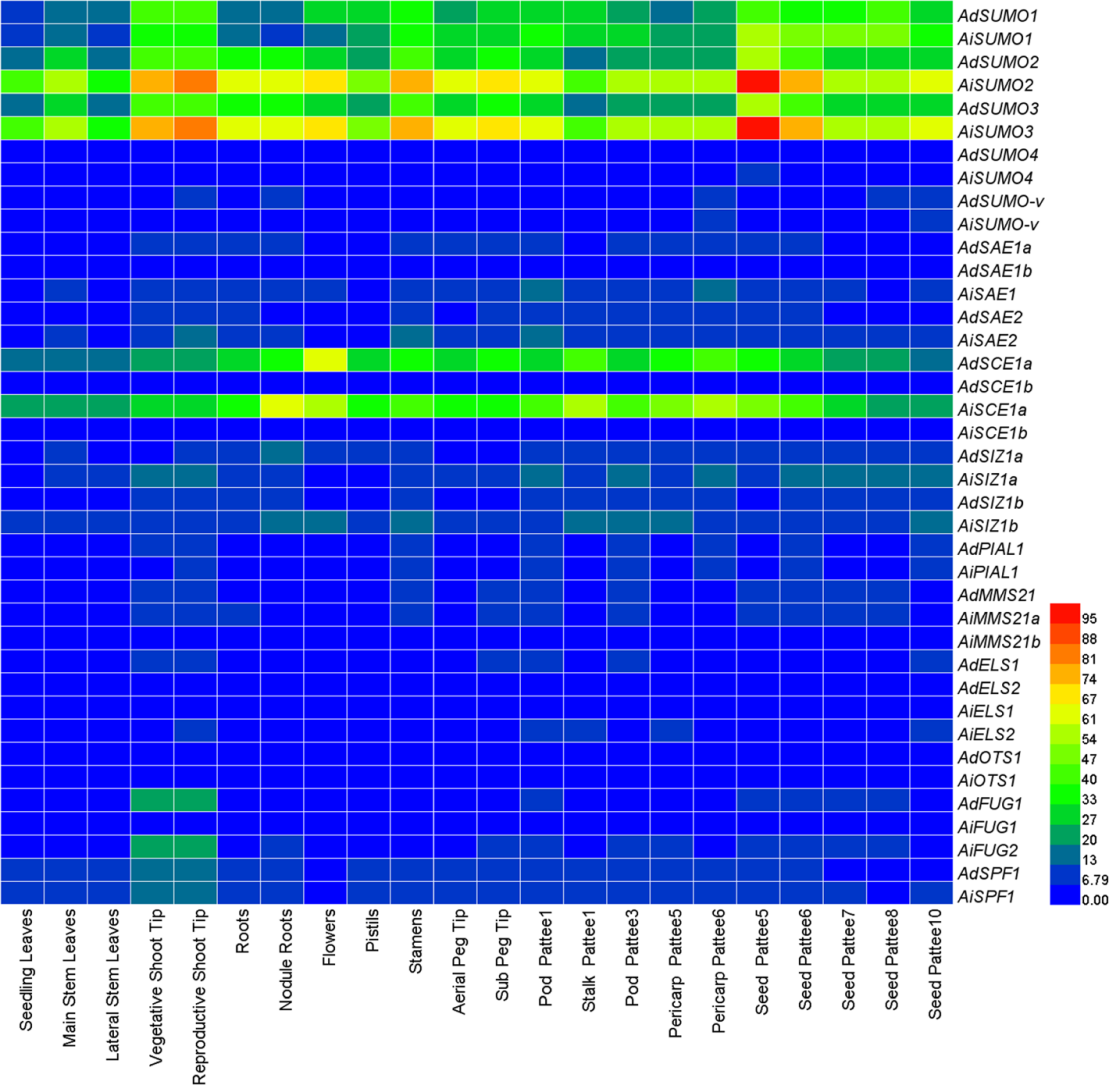
Transcriptome analysis of peanut SUMO system components in different tissues. RNA-seq experiments representing 22 developmentally distinct tissues [48] were mined by reads per kilobase per million reads (RPKM) values for individual SUMO pathway genes. Sub, subterranean

The RPKM data from the RNA-seq of all the SUMO pathway genes in the 22 peanut tissues is shown in Additional file 10: Table S2, implying that SUMOylation is essential to all type tissues of peanut throughout their development. Particularly, *AdSUMO1*/*2/3* and *AiSUMO1 /2/3* were constitutively produced at a relatively high level in all 22 samples, suggesting that these genes might perform a variety of functions at multiple developmental stages in peanut. Notably, SUMO genes showed preferential tissue-specific expression, especially during pod development, suggesting that they may play regulatory roles in the pod development. However, *SUMO4* and *SUMO-v* transcripts were poorly detected throughout the data sets (Fig. 5). In E2 group, *AdSCE1a* and *AiSCE1a* exhibited moderate levels of expression in all of these 22 tissues while *AdSCE1b* and *AiSCE1b* were expressed at low levels during almost all developmental stages, suggesting *SCE1a* may support normal growth and development in peanut. Furthermore, the *MMS21* and *PIAL* genes were poorly expressed in nearly all tissues, while the expression levels of *SIZ1* genes were tissue-specific. For instance, *AiSIZ1a* showed a higher expression in shoot tips and pod development, but *AiSIZ1b* expressed at relatively higher levels in root, nodule, pod and the pericarp development stage (Fig. 5). On the contrary, *AdSIZ1a/b* were hardly detected in any of the investigated tissues. The *ULP* family genes expressed at low levels in almost all samples, except that *Ad/AiFUG1* and *Ad/AiSPF1* were expressed relatively higher in the vegetative and the reproductive shoot tip (Fig. 5).

### SUMOylation profiles in peanut pod development

**Fig. 6:**
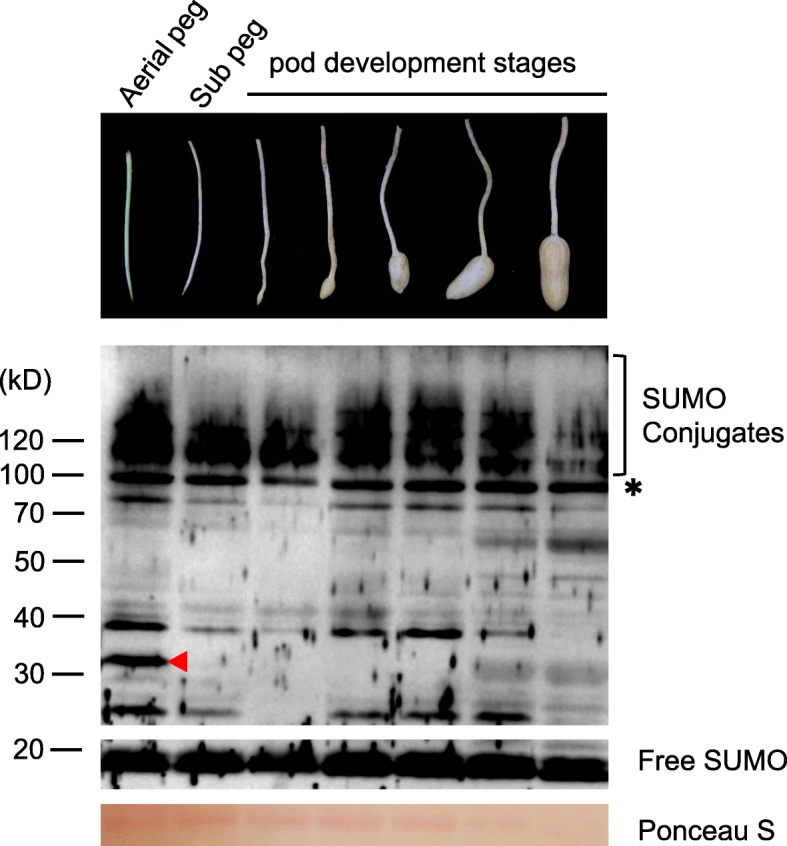
SUMOylation profiles among peanut pod development. Total protein extracts were subjected to immunoblot analysis with anti-AtSUMO1 from various tissues including aerial pegs, subterranean no-swelling pegs and pods of five distinct developmental stages. SUMO conjugates are highlighted by the brackets. The red arrowheads highlight specific SUMO conjugates Ponceau S stained of high abundant protein showed equal loading of protein samples. The asterisk denotes a non-inducible immunoreactive product. Sub, subterranean

As the *SUMO* and *SCE1* genes show higher levels of expression during the peanut pod developmental stage based on RNA-seq data, we detected SUMO conjugates in two stages of pegs (aerial peg and subterranean no-swelling peg) and five distinct stages of pod development. Compared to the aerial pegs, the SUMO conjugates showed reduction in the subterranean no-swelling pegs (Fig. 6). Also, several signature conjugates were not found in the subterranean no-swelling pegs (Fig. 6). After the soil penetration of the peg, the amount of SUMO conjugates sharply decreased, which might have resulted from the darkness and mechanical stimuli. During pod development, the SUMO conjugate profiles showed a gradual rise during seed expansion stages, then decreased as pod maturation (Fig. 6). These results suggested that SUMOylation plays an active role in promoting pod development.

### Stress-regulated SUMOylation in Peanut

**Fig. 7:**
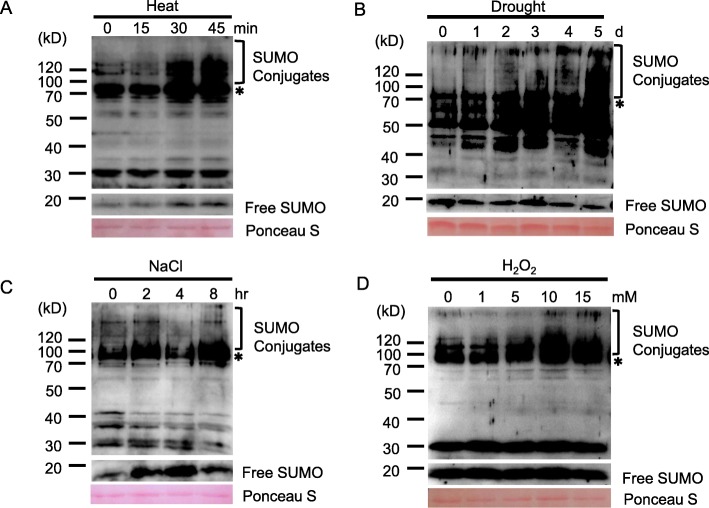
SUMOylation profiles in response to abiotic stress in peanut. Crude extracts from 10-day old leaves were subjected to immunoblot analyses with anti-AtSUMO1. **a** SUMO conjugates of peanut seedlings exposed to 42 °C for 0, 15, 30, 45 min. **b** SUMO conjugates of peanut seedlings treated with drought stress for 0, 1, 2, 3, 4, 5 days. **c** SUMO conjugates of peanut seedlings exposed to 300 mM NaCl for 0, 2, 4, 8 h. **d** SUMOylation is induced by varying concentrations (0, 1, 5, 10, 15 mM) of hydrogen peroxide (H_2_O_2_) for 30 min. SUMO conjugates are highlighted by the brackets. The asterisk denotes a non-inducible immunoreactive product. Ponceau S stained of high abundant protein was included to confirm nearly equal protein loading

Abiotic stresses, such as heat shock, drought, and oxidative stress, have been reported to trigger a significant increase in the SUMO conjugate levels [22, 27, 28, 39]. To better define the SUMOylation response to abiotic stresses in peanut, variation in the SUMO conjugate was investigated in response to heat, drought, salt and hydrogen peroxide (H_2_O_2_). During heat treatment (37 °C), the amount of SUMO conjugates in the peanut leaves remained unchanged after 15 min heat stimulus as compared to the unstressed conditions (Fig.7a). However, the conjugate levels rose substantially from 30 to 45 min after application of heat stress. Also, the level of free SUMO also increased during the treatment at the same time. The results suggested that SUMOylation was involved in the response to high temperature induced stress.

Drought and salt stresses are the major factors impacting on crop yields worldwide [49, 50]. It is increasingly important to study plant adaptation mechanisms to drought and high salinity in soils [20]. In Arabidopsis, it is well established that the abundance of SUMO conjugates increases in response to exposure drought and high salinity [1, 51], but little is known in peanut. So, we first investigated SUMO conjugates of peanut seedlings under drought stress. The amount of SUMO conjugates increased gradually over the increase of the duration of exposure to drought, and culminated after 5 days of drought stress (Fig. 7b). During salt treatment, the accumulation of high molecular weight SUMOylated products was detected after 2 h of salt treatment (Fig. 7c). When treated for 4 h, most free SUMO has been refilled and the amount of SUMO conjugates was greatly reduced. But SUMOylation by salinity treatment at 8 h was rapidly inducible again (Fig. 7c).

Previous reports have shown that the intracellular levels of SUMO conjugates increase on exposure to oxidative stress (H_2_O_2_) in animals and Arabidopsis, so we tested if SUMOs play a comparable role in peanut. Peanut seedlings were exposed for 30 min to various concentrations of hydrogen peroxide (H_2_O_2_). It was observed that significant amount of SUMO conjugates was gradually accumulated with increasing concentrations of H_2_O_2_ (Fig. 7d), which was consistent with Arabidopsis and other plants responding to oxidative stress [28, 43].

## Discussion

In plants, the SUMOylation pathway has significant implications in plant development and their response to environmental stimuli [22]. In this study, 40 SUMO pathway genes in peanut were identified. The peanut genome contained the genes required for SUMOylation that were evolutionarily related to homologs in Arabidopsis and Soybean. On comparison with the eight and six putative SUMO copies found in Arabidopsis and Soybean, respectively; the peanut genome contained eight members sharing a similar ubiquitin-like structure. In the peanut genome, six canonical *SUMO* genes (*AdSUMO1/2/3* and *AiSUMO1/2/3*) were present that homologous to *AtSUMO1/2*. These genes are known conjugate to a variety of target proteins in response to the environmental stimuli [29, 30]. However, the other two genes *Ad/AiSUMO4* were closely related to *AtSUMO5* which belongs to the noncanonical SUMOs. No gene homologs were identified for *AtSUMO3* in the peanut genome. It was suggested that the noncanonical SUMOs evolved multiple times independently followed by rapid diversification [52]. In Arabidopsis, *SUMO3* and *SUMO5*, likely evolved independently from a tandem duplication of *SUMO2* and from an ancient pan-eudicot paleohexaploidy event, respectively [52]. Moreover, *SUMO3* is frequently deleted, converted back to *SUMO2* or pseudogenized in other *Brassicaceae* [43, 52]. This result is in line with the suggestion that, the SUMO copy number of the SUMO genes reverted to a singleton state in plants, and the retained archetype SUMOs have sub-functionalized in terms of their expression pattern and not in terms of their sequence [52]. In addition, plant genomes universally encode a SUMO relative, *SUMO-v*, which is characterized by a long, possibly flexible extension N terminal to the core β-grasp fold [43]. The *SUMO-v* genes in peanut exhibited low expression in the RNA-seq data. These results implicated that six canonical *SUMO* genes in peanut were likely to play crucial roles in plant development. Also, the noncanonical group of *SUMO* genes and *SUMO-v* genes may have other special functions.

There are five *SCE* gene copies that share a conserved UBC domain in peanut, divided into two isotypes. Our results found that peanut also encodes a new type (class II isotype) of the *SCE1*, which previously found to be uniquely present in monocotyledon, such as maize [43]. Sequence analysis and prediction of protein structure have shown that the class II E2s have a more negative electrostatic surface around the active-site Cys which was generated by numerous nonconservative substitutions in addition to an amino acid deletion near the active-site pocket. All these features were consistent with the class II E2s in maize, implying that peanut and maize may have the same evolutionary route. The class I E2s displayed widely expression patterns similar to the canonical SUMOs in the peanut. This indicated that this family of genes cooperated with *SUMOs* play a potentially important role in normal growth and development. By contrast, the class II E2s, *Ad/AiSCE1b*, displayed very low levels of expression in peanut. Previous research on maize had suggested that class II isotype *SCE1f* could display more restricted expression patterns and could effectively directed the SUMOylation of *SUMO1a* [43]. Furthermore, class II SCE1 gene copies was assumed to be co-evolved with certain SUL genes and have composed novel conjugation pathways [43, 53]. Thus, it will be interesting to examine the evolution of class II *SCE1* copies and their catalytic activity to generate poly-SUMO chains.

Additionally, peanut genome encoded three types of SUMO E3 ligases and four types of SUMO proteases. The SUMO E3 ligases are extensively involved in the regulation of environmental stress responses and developmental processes [9, 54]. The expression levels of *SIZ1* genes showed differential tissue specificity. For example, *AiSIZ1a* showed higher levels of expression in the shoot tips, but *AiSIZ1b* expressed relatively higher levels of expression in the root, nodule, and during pod development (Fig. 5). Previous reports in Arabidopsis have found that MMS21 plays a more important role in the DNA damage repair and root development [54, 55]. However, *MMS21* genes were expressed poorly in nearly all tissues, indicating that the role of *MMS21* in peanut is mainly possibly focused on DNA repair. SUMO proteases, which have deSUMOylation activity, also play important regulatory roles in plant development and stress responses [51, 56]. For example, *ESD4* was found to participate in the regulation of flowering of Arabidopsis [57]. *AtSPF1/2* regulate female/male gamete and embryonic development [10, 11], and *OTS1/2* regulate the growth and salt stress responses in rice [12, 24]. The SUMO protease family genes had low levels of expression during in almost all developmental stages, except *Ad/AiFUG1* and *Ad/AiSPF1* were relatively higher expressed in shoot tip, suggesting their potential roles in shoot tip development. Furthermore, the coincident expression of *AiSIZ1a* with *Ad/AiFUG1* and *Ad/AiSPF1* in the shoot tip also raised the possibility that FUG1 and SPF1 may help in the reversal of SIZ1a-mediated SUMOylation.

Biased expression of homoeologous genes in the allopolyploids is considered to be an important feature of polyploidization [58, 59]. The expression of homoeologous SUMO system gene pairs from the A and B subgenomes were also investigated in diverse tissues and developmental stages. Overall, most of the SUMO system homoeologous genes exhibited balanced patterns of expression, which implied that these orthologous genes exhibited functional redundancy. However, some genes from the B subgenome exhibited higher expression level than their homeologs of the A subgenome in some specific tissues and developmental stages. For example, the average *AiSUMO2/3* copies in B subgenome expression level was higher than that of the A subgenome (*AdSUMO2/3*) copies across whole developmental stages. In Shoot tip and seed development stages, *AiSIZ1a* showed higher expression level than *AdSIZ1a* (Fig. 5). The preferential expression might be attributed to the fact that the A and B subgenomes differ in their number and distribution of transposable elements (TEs), with TEs in one genome being closer to genes than they are in the other co-resident genome [60].

The gravitropic growth of the peanut gynophore is one of the most critical characteristics for peanut fructification, in which the peg, a specialized organ that transitions from upward growth habit to downward outgrowth upon fertilization, drives the developing pod into the soil for subsequent development underground [37, 61, 62]. It is still unknown why the aerially developing pod cannot swell normally as compared to that penetrate into soil, although a series of studies attempt to reveal this phenomenon through transcriptomics and proteomics [37, 61, 63]. Our results found that there was no significant difference in the expression level of SUMO pathway genes between the aerial peg stage and the subterranean peg stage. However, the amount of SUMO conjugates decreased sharply, especially that several SUMO conjugates disappeared after the peg has penetrated into the soil (Fig. 6). It was thought that the elongation and swollen of the peanut peg was affected by auxin, gibberellin, and other phytohormones [35, 62, 64]. On the other hand, assessment of the SUMOylome revealed that SUMOylation participates in the transport and signaling of plant hormone (auxin, gibberellin, and abscisic acid). These results implied SUMOylation may control peg elongation and swollen through the plant hormone pathway at post-translational level. Further efforts to identify SUMO substrates specially involved in this process will contribute to the understanding of this regulation mechanism in peanut.

Although transcriptional analysis showed that the SUMO system was essential to most tissue types, we noticed an intimate connection specifically with pod development. In peanut, seed expansion began at Pattee stage 5 (early pod development stage), where sugar content reaches a maximum in the pericarp and the seed begins to differentiate from the pericarp [65]. At seed Pattee 5 stage and continuing into stage 6, all canonical SUMO genes were highest expressed and then as seed development progressed their expression decreased (Fig. 5). Meanwhile, the SUMO conjugations also rose strongly at stage 5 during early pod development, and then decreased (Fig.6), thus raising the possibility that increased SUMO abundance drives increased SUMOylation. Previous developmental transcriptome of peanut showed that top enriched genes in this stage include mitosis, DNA methylation, microtubule-based movement, glycolysis, and fatty acid biosynthesis [48]. Coincidently, proteome-wide identification of the SUMO substrates also detected a significant enrichment in proteins involved in the DNA modification, catabolism, and microtubule-based movement [30]. Given that the development of SUMO conjugation coincided with the increased expression of canonical *SUMOs*, it is possible that, during seed expansion, the transcripts of *SUMO* genes would be activated first and then SUMOylation would modify the substrates related to mitosis, DNA methylation, microtubule-based movement, and fatty acid biosynthesis to regulate the early pod development.

A variety of abiotic stresses are known to cause the accumulation of SUMO conjugates, including oxidative stress, salt, drought, and temperature stresses [1, 20, 23]. Despite the well characterization of the SUMOylation-dependent abiotic stress responses in Arabidopsis, there is growing evidence that this link is conserved in many crop species such as rice, maize or soybean [41–44]. For example, the levels of SUMO conjugates increase in response to abiotic stress such as cold, high salinity or increased ABA in rice [44], and in response to heat and oxidative stress in maize [43]. Moreover, the levels of SUMO conjugates also increase in the soybean plants that are exposed to various abiotic stresses including high salinity, heat or increased ABA [41, 42]. Our results showed that SUMO conjugates on protein level increased initially and then decreased, when seedlings subjected to salt stresses. Moreover, peanut is considered as a moderately salt-sensitive species [39, 40]. This result implied that the responses to salt stresses were adapting in peanut. It was important to note that the SUMO conjugates gradually accumulated in response to drought, heat and H_2_O_2_ treatments in peanut (Fig.7). The investigation of SUMOylation profiles demonstrated that peanut had conserved responses to drought, high temperature and oxidative stresses by the regulation of the SUMO conjugated proteins like other plants. These results revealed the complexity of post-translational modifications of SUMOylation in response to environmental stresses in peanut.

## Conclusion

In summary, a total of 40 genes involved in SUMOylation system were identified from the peanut genome. The characterization and tissue-specific expression profiles of the SUMO pathway genes implied that the SUMO pathway members might play a role in various tissues in peanut. Moreover, the immunoblot analysis demonstrated that SUMOylation could be involved in abiotic stresses by protein modifications and could play a key role during pod development. Given the heightened SUMOylation seen during stress and pod development, further proteome-wide screens for identifying SUMO substrates will provide new strategies to enhance agronomic yield and reveal the mechanism of peanut pod development.

## Methods

### Identification of Peanut SUMO pathway genes

SUMO pathway genes in peanut were identified using the known pathway components from Arabidopsis (*Arabidopsis thaliana*) ecotype Columbia-0 as queries. The SUMO pathway protein sequences were downloaded from Arabidopsis Information Resource website (TAIR, http://www.arabidopsis.org). BLASTP and TBLASTN analyses were performed with two wild peanut species (AA and BB genomes) available in the peanut genome database (http://peanutbase.org/). Reciprocal BLASTP analysis was performed using NCBI to ensure that the subject hits most closely matched the SUMO pathway query. Protein domains were predicted using Pfam database (http://pfam.xfam.org). The protein sequences were collected from the related genome databases for the following additional plant species: Maize genome database (https://maizegdb.org/), Soybean genome database (https://soybase.org/soyseq/), *Oryza sativa* genome database (http://www.plantgdb.org/OsGDB/). The amino acid sequences of the SUMO pathway components in this study were shown in Additional file 9: Table S1. Possible SUMOylation sites and SIM sequences were predicted using the default settings in GPS-SUMO version 1.0.1 [45].

### Multiple sequence alignment, gene structure and phylogenetic analysis

Protein multiple sequence alignment was performed using software Clustal X 2.0 [66] and the alignment was edited with GeneDoc (http://www.psc.edu/biomed/genedoc/). Furthermore, the Gene Structure Display Server 2.0 (GSDS) was used to draw the gene structure of SUMO system genes [67]. The phylogenetic trees were constructed using MEGA 5 with protein sequences, applying the neighbor-joining (NJ) method with a bootstrap test of 1000 replications and complete deletion of the gaps [68].

### Protein model threading

For three-dimensional structure predictions, the *Arachis ipaensis* SCE1a/c protein sequences were threaded by SWISS-MODEL (http://swissmodel.expasy.org [69];) into the crystallographic structure of human UBC9. Threaded PDB outputs were visualized in PyMOL version 1.7.0.3 (http://www.pymol.org). The Adaptive Poisson Bolzmann Solver plugin version 2.1 calculated electrostatic surface densities.

### Detection of orthologous gene pairs and Synteny analysis

MCScanX v0.8 software [70] (http://chibba.pgml.uga.edu/mcscan2/) was used to detect the duplicated genes within peanut genome and the syntenic blocks among peanut and sorghum. Whole-genome protein sequences from peanut and sorghum were merged and searched against themselves using BLASTP with an E-value cutoff of 1 × 10^− 5^, then, the default parameters of MCScanX and associated downstream tools. The position of each SUMO genes was marked on the chromosomes using a Perl script. The relationships of the orthologous pairs among the two species were plotted using Circos (http://circos.ca/) [71].

### Plant growth and treatments

The peanut cultivar Luhua 14 (LH14), cultivated by colleagues in the Shandong Institution of Peanut (http://www.sdshss.com/) [39], was used as the experimental materials in this study. After germination in sand for 8 days, peanut seedlings were transferred to hydroponic pots containing 2 L of Hoagland’s nutrient solution and grown in an artificial climate-controlled chamber with 16 h light (200 μmol protons m^− 2^ s^− 1^, 26 °C) and 8 h darkness (24 °C) at 50% relative humidity. The nutrient solution was changed weekly. Treatments began when seedlings were 18 days old. Each containing four replicate plants in separate pots. For NaCl treatments, seedlings were grown in nutrient solution with addition of 300 mM NaCl for 0, 2, 4, and 8 h. For heat treatment, seedlings were transferred into a 42 °C growth chamber for 0, 15, 30, and 45 min. For H_2_O_2_ treatment, peanut plants were exposed for 30 min to various concentrations (0, 1, 5, 10, 15 mM) of hydrogen peroxide (H_2_O_2_). For the drought stress conditions, peanut seeds were germinated and grown in sand for 16 days with 16 h light (200 μmol protons m^− 2^ s^− 1^, 26 °C) and 8 h darkness (24 °C) at 50% relative humidity. During this period, seedlings were watered every 4 days. Then the 16-old-day seedlings were subjected to drought by withholding water for 0, 1, 2, 3, 4, 5 days. At the end of the experiments, all leaves were collected, immediately frozen in liquid nitrogen, and stored at − 80 °C until analysis. For the analysis of peanut pegs development, seven developmental stages of peanut pegs were used in this study. Aerial grown pegs, which were green or purple in color with a length of 3–5 cm; peg grown in soil for about 3 d with no detectable ovary enlargement; pod development for about 15 d, 30 d, 45 d, and 60 d respectively. A tip region of the peg was manually dissected, frozen in liquid nitrogen and stored at − 80 °C for the following experiments.

### RNA-seq data and expression of SUMO pathway genes

To further characterize the function of peanut SUMO pathway genes during peanut development, RNA-seq data from 22 different tissues in cultivated peanut were downloaded from the National Center for Biotechnology Information (http://www.ncbi.nlm.nih.gov/) under BioProject PRJNA291488 [48]. A description of the peanut tissues is listed in Additional file 10: Table S2. The expression pattern of the SUMO Pathway genes in different tissues was generated with Heml 1.0 heatmap illustrator [72].

### Protein extraction and Immunoblotting analysis of SUMO conjugates

Total protein from peanut different tissues was isolated using Plant Active Protein Extraction Kit (Sangon Biotech, Shanghai, China) according to manufacturer’s instructions. Protein concentration was determined by Bradford method using BSA as the standard. 20 μg of purified protein were separated on 12% SDS − PAGE and transferred to nitrocellulose membrane according to standard protocols. Gel loading control was done by Ponceau S staining (right after transfer). After washing by TBST buffer (10 mMTris-HC1, pH 8.0, 150 mM NaCl, 0.05% Tween 20), the membrane blocked in 5% dried milk in TBST buffer for 1 h at room temperature, then probed with AtSUMO1 primary antibody (Abcam, China, ab5316) which was prepared in the TBST buffer containing 1% milk (1:2000) at 4 °C for overnight. After removing unbound antibodies by washing with a TBST buffer, the blot was incubated with goat anti-rabbit IgG secondary antibody (horseradish peroxidase conjugated, Thermo Fisher Scientific, USA) in the TBST buffer at a dilution of 1:5000. The samples were detected with the ECL Plus kit according to manufacturer’s (Merck Millipore, USA) instructions.

## Supplementary information

**Additional file 1: Figure S1.** Gene structure of SUMO System genes in peanut. The blue boxes indicate the exons while the single lines indicate introns. Gene models were drawn to scale as indicated at the bottom.

**Additional file 2: Figure S2.** Sequence alignment of SUMOs from peanut. AdSUMO1/2/3/4, AiSUMO1/2/3/4, AdSUMO-v and AiSUMO-v sequence alignment was performed using software Clustal X 2.0 and the alignment was edited with GeneDoc. Gray and black boxes identify similar and identical amino acids, respectively. Dashes denote gaps.

**Additional file 3: Figure S3.** Sequence alignment of SAEs from peanut and other species. SAEs (E1) protein sequences from *Arabidopsis thaliana*, *Zea mays*, *Oryza sativa*, peanut and *Glycine max* were used to construct the phylogenetic tree by the neighbor-joining method in MEGA 5.

**Additional file 4: Figure S4.** Sequences alignment analysis between AdSCE1a and AiSCE1a.

**Additional file 5: Figure S5.** Alignment of SUMO ligase SIZ1 type protein sequences. SAP, PHD, MIZ/SP-RING domains, PINIT and SXS motifs are indicated above the sequence by blue lines. Residue numbers are shown for each polypeptide. Grey and black boxes identify similar and conserved amino acids, respectively. Dashes denote gaps.

**Additional file 6: Figure S6.** Alignment of SUMO ligase PIAL type protein sequences. MIZ/SP-RING domains and SIMs are indicated above the sequence by blue lines. Residue numbers are shown for each polypeptide. Grey and black boxes identify similar and conserved amino acids, respectively. Dashes denote gaps.

**Additional file 7: Figure S7.** Alignment of SUMO ligase MMS21 type protein sequences. SP-RING domains are indicated above the sequence by blue bare. Residue numbers are shown for each polypeptide. Grey and black boxes identify similar and conserved amino acids, respectively. Dashes denote gaps.

**Additional file 8: Figure S8.** Sequence alignment of the SUMO protease family in peanut. The extent of the C48 Peptidase domain is indicated above the sequence alignment by blue lines. Residue numbers are shown for each polypeptide. Grey and black boxes identify similar and conserved amino acids, respectively. Dashes denote gaps.

**Additional file 9: Table S1.** The amino acid sequences of the SUMO pathway components in this study.

**Additional file 10: Table S2.** RNA-Seq data of SUMO pathway genes in peanut.

## Data Availability

The materials used during the current study are available from the corresponding authors on reasonable request.

## Abbreviations

Cys: Cysteine
RPKM: Reads Per Kilobase per Million mapped reads
SAE: SUMO activating enzymes
SCE: SUMO-conjugating enzyme
SUMO: Small ubiquitin-related modifier
ULP: Ubiquitin-like protein protease
