# Supplementary figures and images for: Defining the function of SUMO system in pod development and abiotic stresses in Peanut
Source: BMC Plant Biol. 2019 Dec 29;19:593. doi: 10.1186/s12870-019-2136-9 (PMC7194008; doi:10.1186/s12870-019-2136-9)

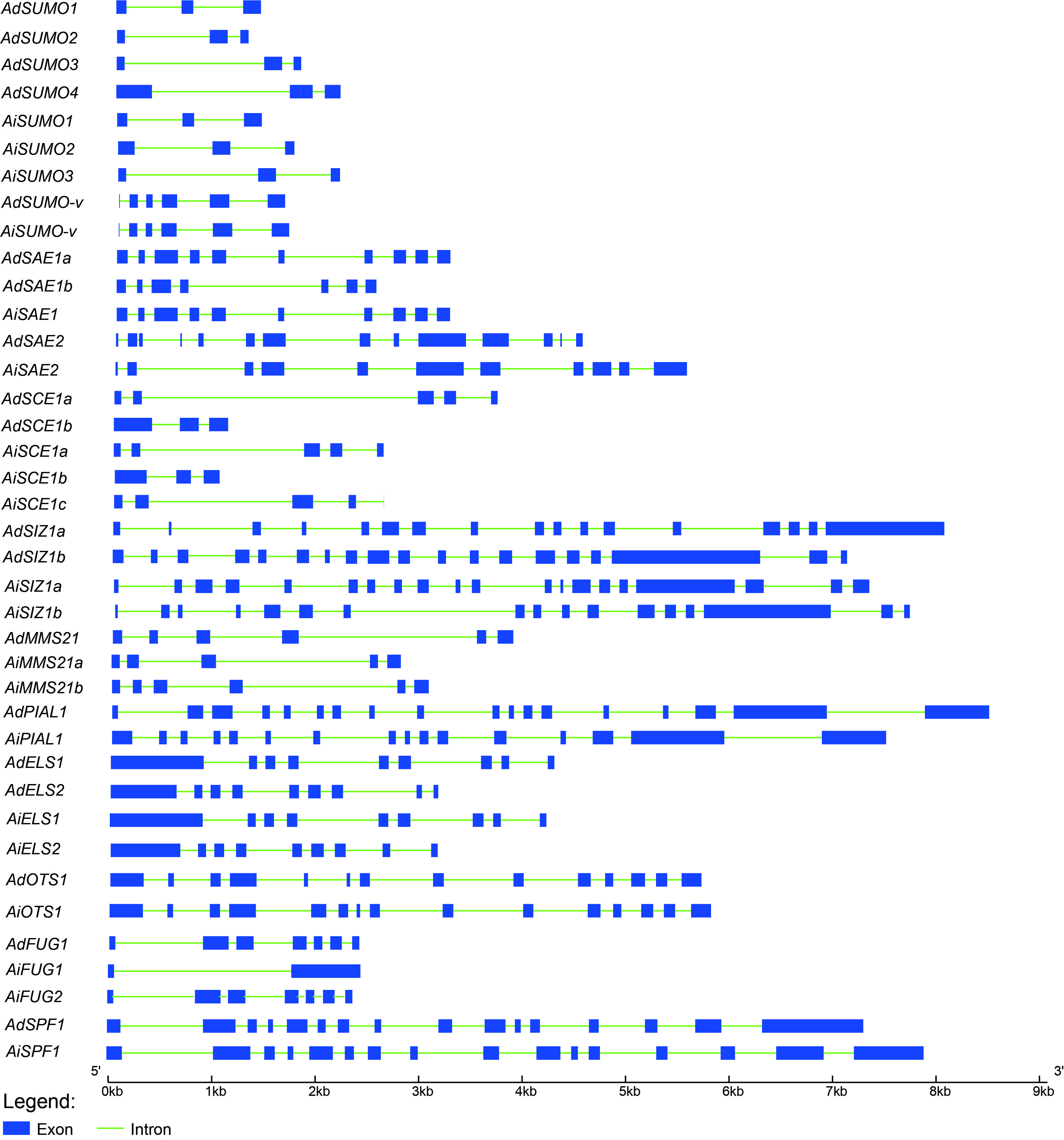

Supplement: Supplementary file 1 — Additional file 1: Figure S1. Gene structure of SUMO System genes in peanut. The blue boxes indicate the exons while the single lines indicate introns. Gene models were drawn to scale as indicated at the bottom. [file 12870_2019_2136_MOESM1_ESM.jpg]

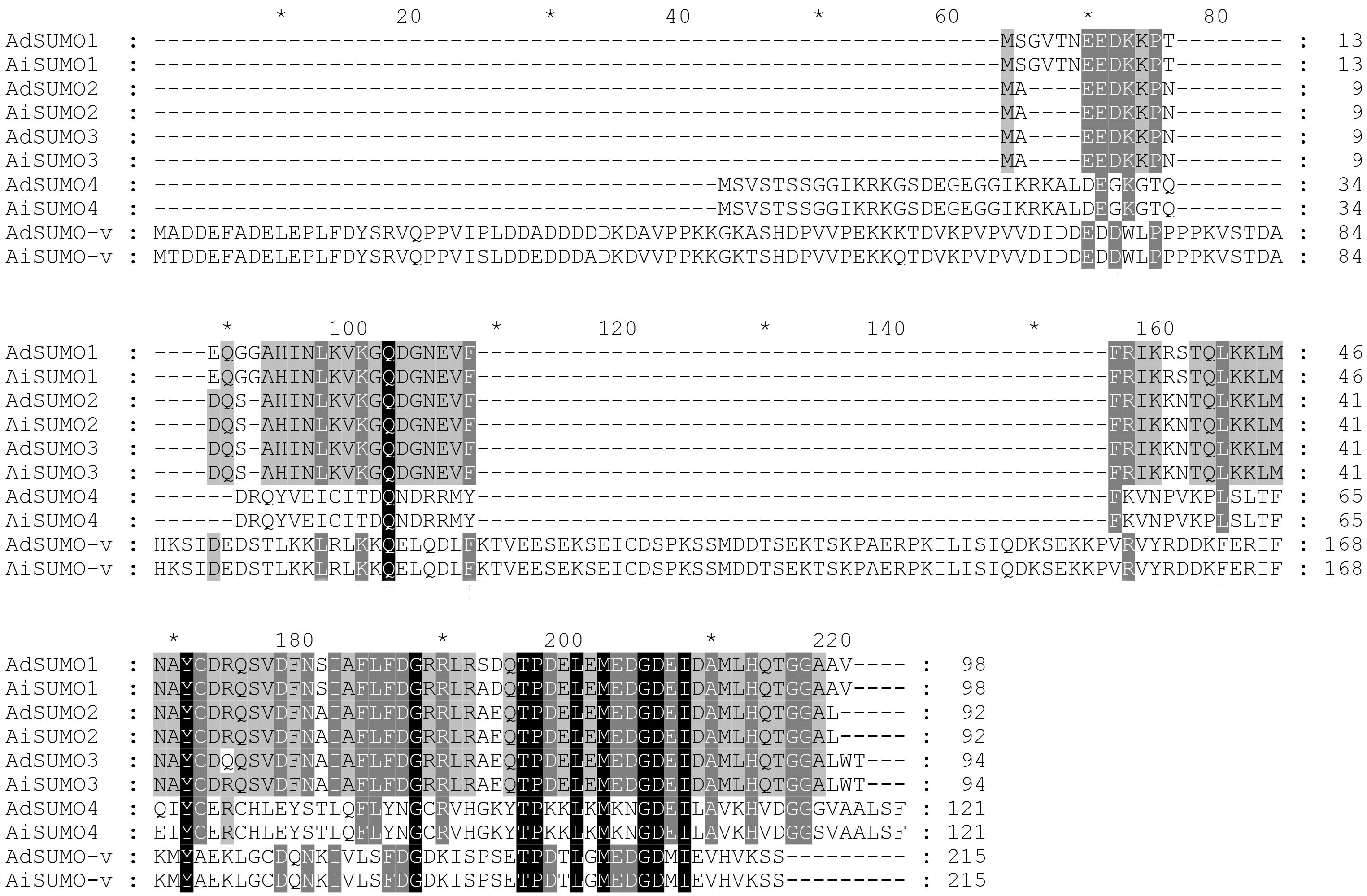

Supplement: Supplementary file 2 — Additional file 2: Figure S2. Sequence alignment of SUMOs from peanut. AdSUMO1/2/3/4, AiSUMO1/2/3/4, AdSUMO-v and AiSUMO-v sequence alignment was performed using software Clustal X 2.0 and the alignment was edited with GeneDoc. Gray and black boxes identify similar and identical amino acids, respectively. Dashes denote gaps. [file 12870_2019_2136_MOESM2_ESM.jpg]

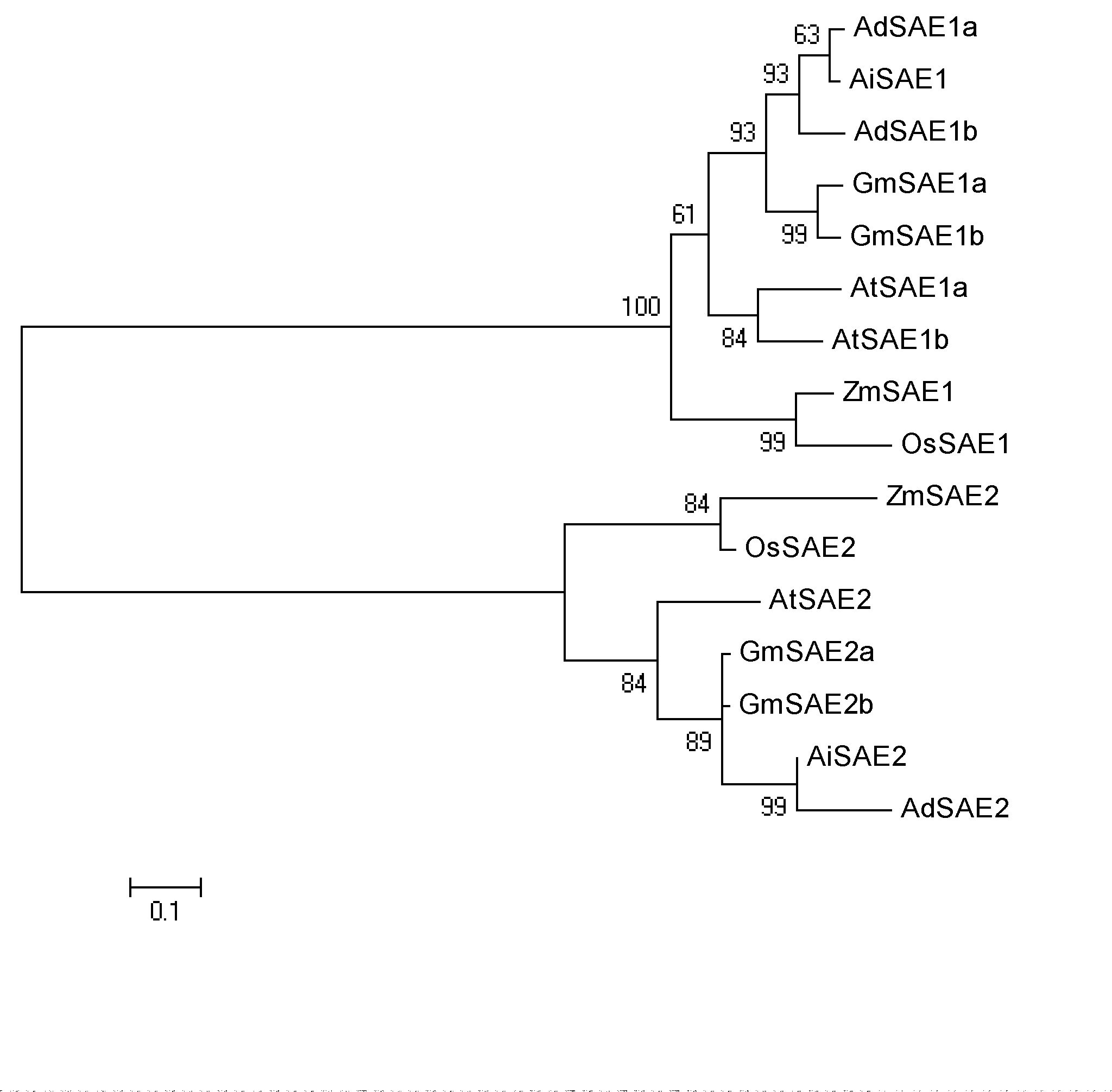

Supplement: Supplementary file 3 — Additional file 3: Figure S3. Sequence alignment of SAEs from peanut and other species. SAEs (E1) protein sequences from Arabidopsis thaliana, Zea mays, Oryza sativa, peanut and Glycine max were used to construct the phylogenetic tree by the neighbor-joining method in MEGA 5. [file 12870_2019_2136_MOESM3_ESM.gif]

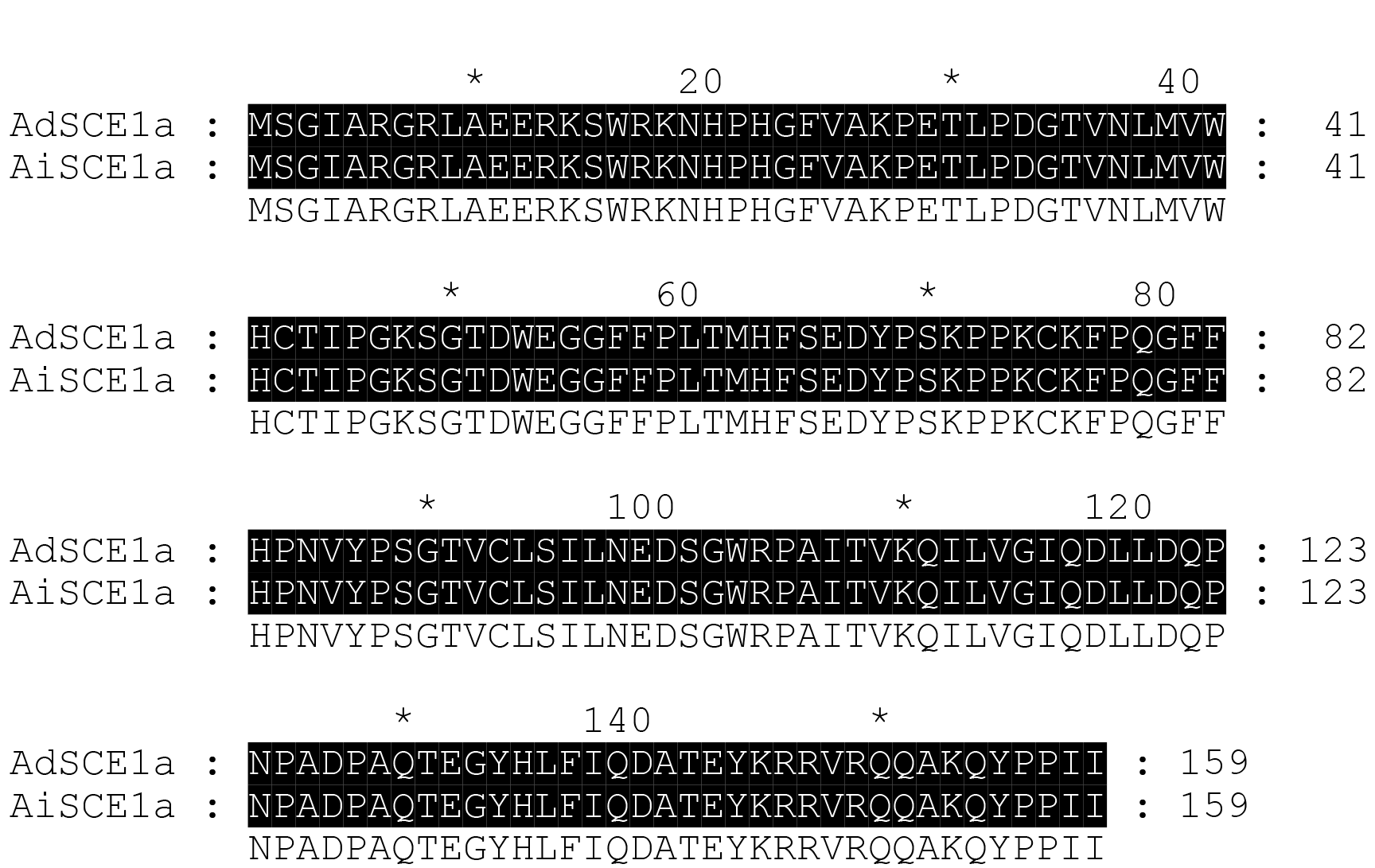

Supplement: Supplementary file 4 — Additional file 4: Figure S4. Sequences alignment analysis between AdSCE1a and AiSCE1a. [file 12870_2019_2136_MOESM4_ESM.tif]

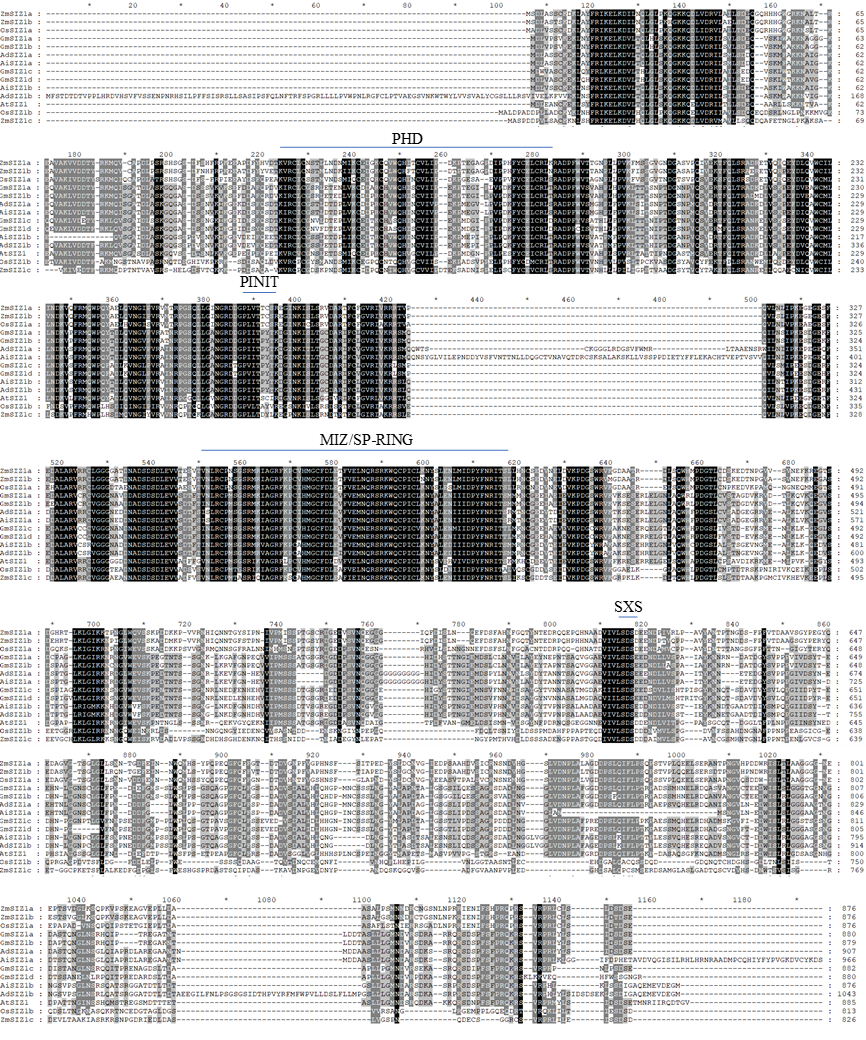

Supplement: Supplementary file 5 — Additional file 5: Figure S5. Alignment of SUMO ligase SIZ1 type protein sequences. SAP, PHD, MIZ/SP-RING domains, PINIT and SXS motifs are indicated above the sequence by blue lines. Residue numbers are shown for each polypeptide. Grey and black boxes identify similar and conserved amino acids, respectively. Dashes denote gaps. [file 12870_2019_2136_MOESM5_ESM.tif]

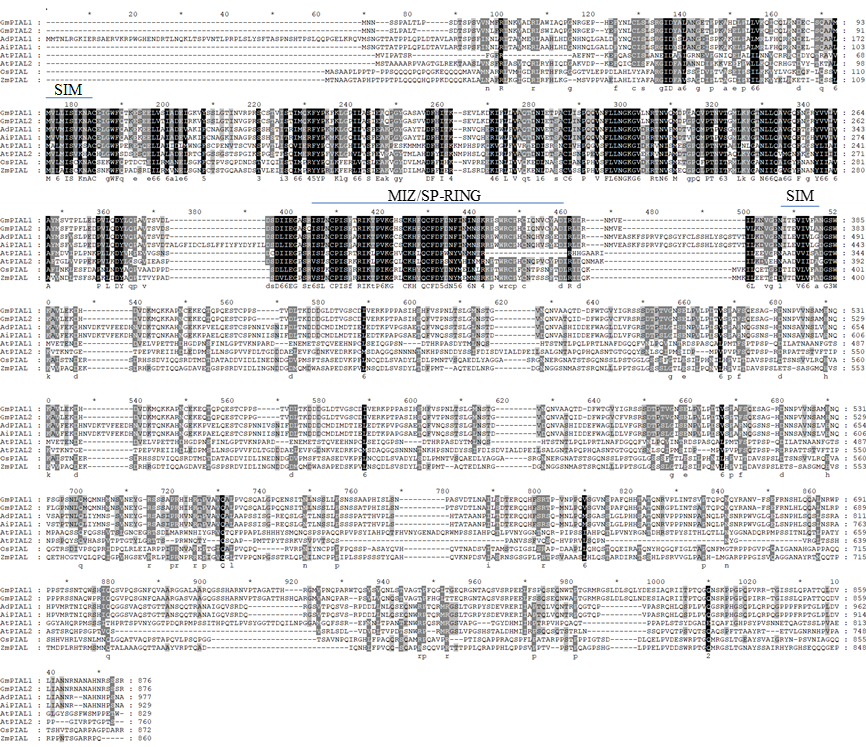

Supplement: Supplementary file 6 — Additional file 6: Figure S6. Alignment of SUMO ligase PIAL type protein sequences. MIZ/SP-RING domains and SIMs are indicated above the sequence by blue lines. Residue numbers are shown for each polypeptide. Grey and black boxes identify similar and conserved amino acids, respectively. Dashes denote gaps. [file 12870_2019_2136_MOESM6_ESM.tif]

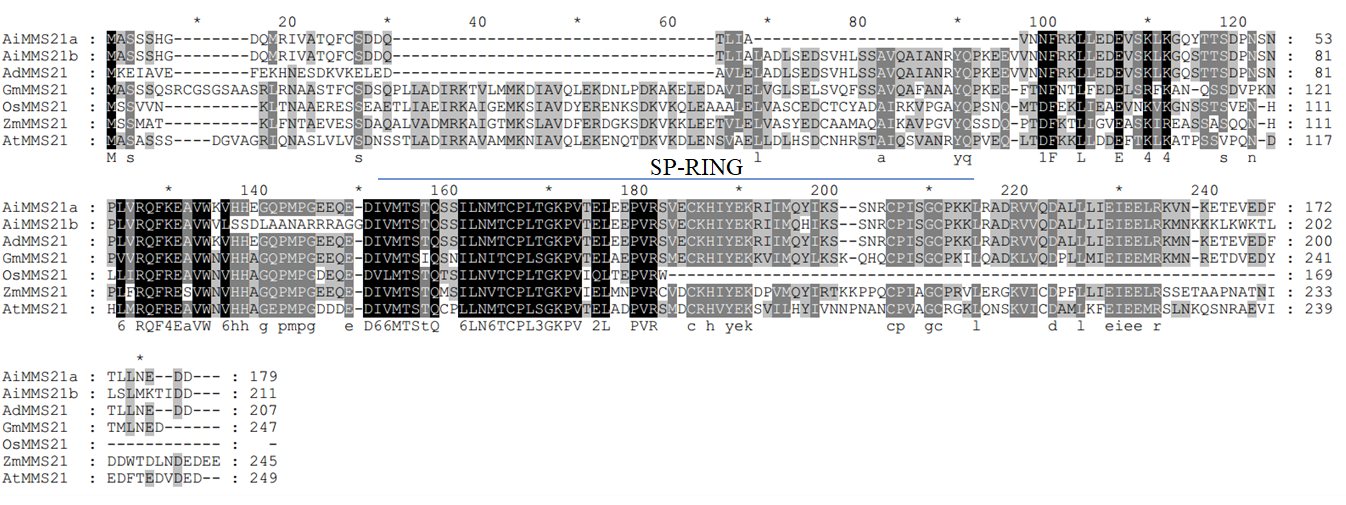

Supplement: Supplementary file 7 — Additional file 7: Figure S7. Alignment of SUMO ligase MMS21 type protein sequences. SP-RING domains are indicated above the sequence by blue bare. Residue numbers are shown for each polypeptide. Grey and black boxes identify similar and conserved amino acids, respectively. Dashes denote gaps. [file 12870_2019_2136_MOESM7_ESM.tif]

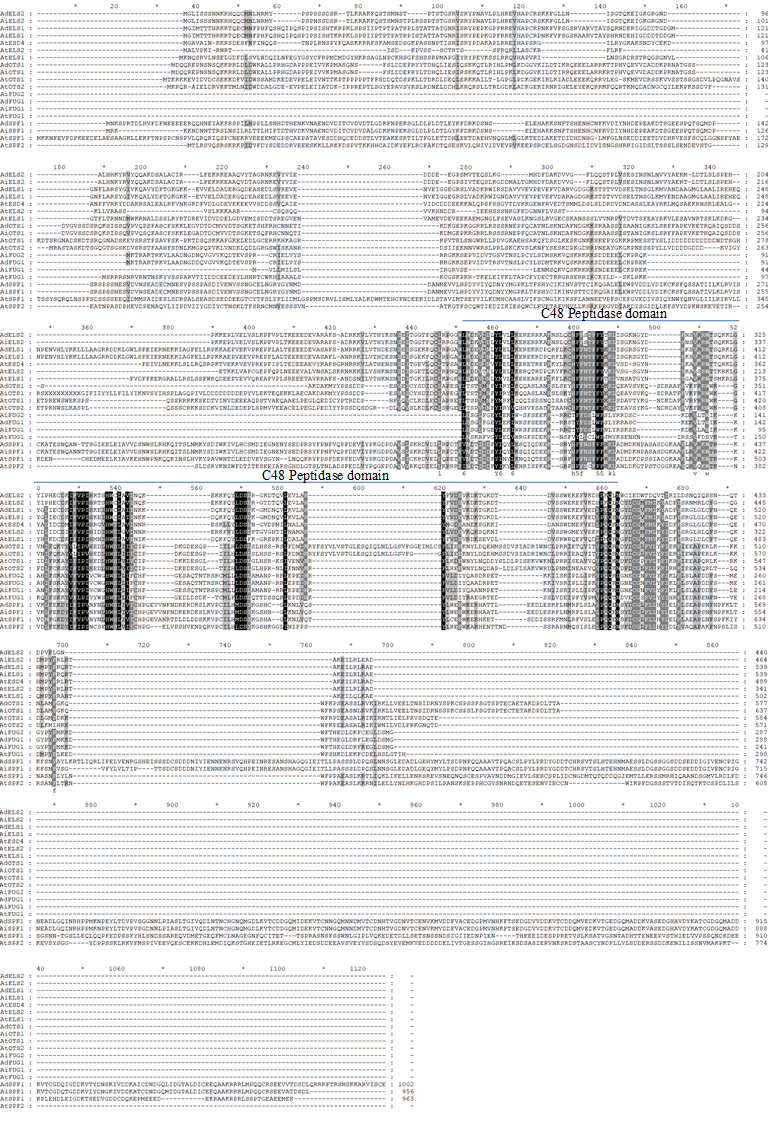

Supplement: Supplementary file 8 — Additional file 8: Figure S8. Sequence alignment of the SUMO protease family in peanut. The extent of the C48 Peptidase domain is indicated above the sequence alignment by blue lines. Residue numbers are shown for each polypeptide. Grey and black boxes identify similar and conserved amino acids, respectively. Dashes denote gaps. [file 12870_2019_2136_MOESM8_ESM.tif]
